# A Testing and Evaluation Framework for Indoor Navigation and Positioning Systems

**DOI:** 10.3390/s25072330

**Published:** 2025-04-06

**Authors:** Zhang Zhang, Qu Wang, Wenfeng Wang, Meijuan Feng, Liangliang Guo

**Affiliations:** 1 China Electronics Standardization Institute, Beijing 100007, China; zhangzhang@cesi.cn (Z.Z.); wangwf@cesi.cn (W.W.); 2 School of Automation Science and Electrical Engineering, University of Science and Technology Beijing, Beijing 100083, China; 3 Shunde Graduate School, University of Science and Technology Beijing, Foshan 528399, China; 4 Department of Energy and Control Engineering, Shanxi Information Industry Technology Research Institute Co., Ltd., Taiyuan 030012, China; fengmeijuan_lucky@163.com (M.F.); x13994219500@126.com (L.G.)

**Keywords:** indoor positioning systems, performance evaluation, testing, standardization, testing and evaluation

## Abstract

The lack of a testing framework for various indoor positioning technologies brings huge challenges to the systematic and fair evaluation of positioning systems, which greatly hinders the development and industrialization of indoor positioning technology. In order to solve this problem, this article refers to international standards, such as ISO/IEC 18305, and uses the China Electronics Standardization Institute’s rich experience in indoor positioning technology research and testing to build a universal positioning performance testing and evaluation framework. First, this paper introduces the experimental environment in detail from the aspects of the coordinate system definition, test point selection, building type definition, motion mode definition, and motion trajectory setting. Then, this paper comprehensively measures performance evaluation indicators from dimensions such as the accuracy index, relative accuracy, startup time, fault tolerance, power consumption, size, and cost. Finally, this paper elaborates on the testing methods and processes of positioning precision, accuracy, relative accuracy, floor identification, indoor–outdoor distinction, latency, relative accuracy, success rate, and movement speed tests.

## 1. Introduction

High-precision indoor location services play a vital role in spatial intelligence and the Internet of Everything, making it a research focus in academia and industry [1,2]. Location services are finding increasing applications across various domains, including public security, counter-terrorism and stability maintenance, emergency response, smart cities, intelligent manufacturing, internet services, and smart transportation [3,4].

As global navigation satellite systems (GNSSs) progress and evolve, satellite-based navigation and positioning have increasingly replaced traditional geodetic and astronomical methods, owing to their capacity to rapidly provide spatial location data in outdoor environments [5]. The GNSS is extensively utilized in pedestrian positioning [6,7,8], vehicle navigation [9,10,11,12,13], deformation monitoring [14,15], and other related fields, fostering innovation and advancement in geodesy and navigation positioning. Currently, smartphones predominantly rely on GNSSs for location tracking in outdoor settings [16]. In open outdoor settings, GNSSs can obtain a positioning accuracy of about 3 m [17]. However, in indoor environments, such as warehouses, buildings, and large venues, it is difficult to complete positioning due to the obstruction of satellite signals and multipath effects [5].

However, people spend more than 80% to 90% of their time indoors. Achieveing precise positioning in complex indoor scenes is the key to achieving seamless indoor and outdoor location services [18,19]. According to a market analysis report by MarketsandMarkets, the indoor positioning market is projected to expand from USD 10.9 billion in 2023 to USD 31.4 billion in 2029, with a compound annual growth rate of 21.4% [20]. Generally speaking, the main application scenarios for indoor positioning technology include hospitals, prisons, airports, large shopping malls, large parking lots, railway stations, exhibition halls, comprehensive office buildings, tourist attractions, underground operations, and other industry fields [21,22,23]. Its functions include indoor positioning and navigation, the monitoring of personnel and equipment, electronic fences, activity analysis, reverse car searches in parking lots, etc.

**Figure 1:**
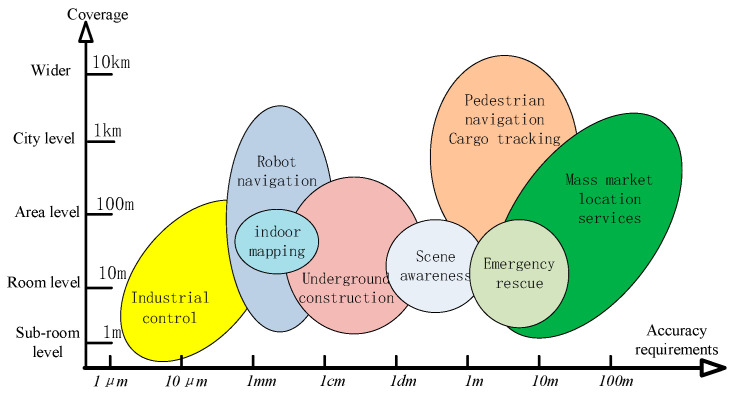
Accuracy requirements of indoor positioning in different fields.

Different fields have different requirements for the location service coverage and accuracy. Figure 1 shows the requirements for the coverage and positioning accuracy in different fields [1]. High-precision navigation and positioning are essential in professional fields. Industrial control [24,25] and indoor surveying and mapping [26] have smaller coverage requirements but require millimeter-level positioning accuracy. Scenarios such as underground or indoor construction [27,28] and robot motion control [29,30] require hundreds of meters of coverage and decimeter-level positioning accuracy. In scenarios such as pedestrian navigation [31] and emergency rescue [32,33], due to the strong autonomous perception ability of people, a positioning accuracy of several meters can meet the needs, but a coverage range of several kilometers is required [34,35].

### 1.1. Existing Indoor Localization Technologies

**Figure 2:**
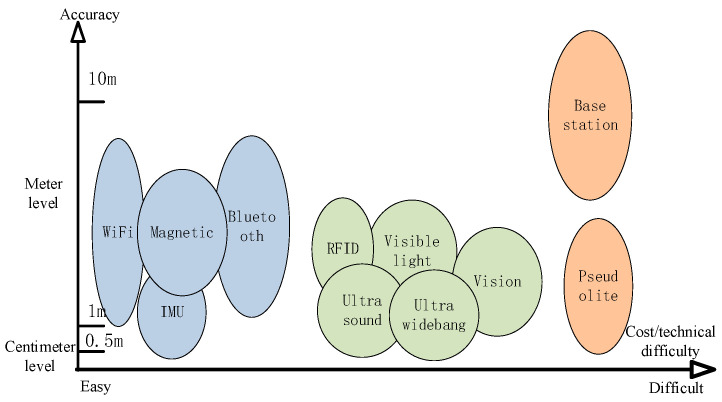
Comparison of accuracy and complexity of different indoor positioning technologies.

In recent years, indoor positioning technologies have expanded to include base station positioning [36,37,38], radio frequency identification (RFID) [39,40], ultra-wideband (UWB) [41,42], Bluetooth low energy (BLE) [43,44], WLAN [45,46,47], ultrasound [48], visible light [49,50,51], vision [52,53,54], geomagnetism [8,55,56,57,58,59], inertial navigation systems (INSs) [60,61,62,63], pseudolites [64,65], and other technologies. Table 1 summarizes the current mainstream indoor positioning technologies regarding the basic principles, representative systems, positioning accuracy, advantages, and disadvantages. Every navigation and positioning technology must make a trade-off between cost and accuracy. Figure 2 summarizes the relationship between the accuracy of these positioning techniques and their ease of implementation. These indoor positioning techniques can be categorized in various ways depending on specific criteria. In terms of usage contexts, indoor positioning technology can be categorized into localized indoor positioning and broad-area indoor positioning [1]. Local indoor positioning technology achieves positioning coverage in a limited area, which is characterized by a short deployment time, low cost, and high positioning accuracy. However, it faces problems such as limited positioning sites and the difficulty in unifying the methods used in the positioning area. Typical local area positioning methods include Wi-Fi positioning, BLE positioning, and ZigBee positioning. Broad-area indoor positioning technology employs unified standards to achieve large-area positioning, generally requiring modifications to receiving terminals and base stations, which is time-consuming and costly. Representative technologies include TC-OFDM [66], Locate [67], Qpoint, and other positioning systems. According to different positioning methods, indoor positioning can be categorized into specific device positioning, wireless signal-based positioning, and mobile sensor-based positioning [1]. Specific device positioning methods utilize terminal devices with signal transceivers, calculations, and other functions specially designed to achieve positioning requirements, such as infrared receivers, ultrasonic receivers, and radio frequency identification (RFID) receivers. These devices receive signals with special identifiers emitted by pre-deployed hardware in the environment, calculating the relative position information between the undetermined position and the known device to determine its absolute position. Existing positioning systems based on specific devices include Active Badge [68], Active Bat [69], Ubisense [70], and LANDMARC [71]. The wireless signal positioning method uses wireless signals that are prevalent in the area to achieve positioning. At present, Wi-Fi routers are frequently installed in indoor settings, like shopping centers, medical facilities, and corporate offices. At the same time, mobile terminals, such as laptops, tablets, and smartphones, have wireless network cards, making wireless positioning methods based on Wi-Fi signals widely used. Mobile sensor-based positioning methods benefit from the rapid development of micro-electro-mechanical systems (MEMSs), which has led to the miniaturization of inertial measurement components. Coupled with the continuous enhancement of the terminal hardware and computing capabilities, mobile-based sensor positioning methods can effectively obtain users’ behavior, movement, and location trajectory information, providing stable positioning results in complex and changeable indoor environments. For example, Finland’s IndoorAtlas [72] system uses inertial sensors and magnetometers for indoor positioning and navigation. Achieving indoor positioning with a positioning accuracy better than 1 m, low cost, and broad-area coverage is still the biggest obstacle facing high-precision indoor and outdoor seamless positioning services.

After more than two decades of development, indoor positioning technology has attained a high degree of sophistication and is beginning to penetrate the market. The performance testing and evaluation of indoor navigation and positioning systems is the core link to ensure technical reliability and application security. The multi-dimensional performance evaluation (such as positioning accuracy, integrity, and continuity) and extreme scenario testing (urban canyons and electromagnetic interference) can reveal the true performance boundaries of positioning systems in complex environments and are also the key basis for optimizing multi-source fusion algorithms (GNSS/INS/vision collaboration). However, different researchers and localization methods use different methods to test and evaluate the proposed localization methods. The marketization of indoor positioning systems requires the accurate quantification of their performance. Only in this way can users evaluate whether the positioning system meets their needs. In addition, researchers or developers of positioning systems need to evaluate their systems to objectively evaluate whether their systems are ready for the market. To better evaluate, researchers try to build standards and competitions to provide a universal and fair performance testing platform.

### 1.2. Related Standards

Standardization is key to enabling code reuse, data sharing, system integration, and performance testing. Furthermore, standardization can reduce the difficulty of cross-system integration and promote collaboration between different research institutions and industries, thereby improving the efficiency of positioning technology research and development. At present, the existing standardization work in the field of navigation and positioning is as follows:

- ISO/IEC 18305:2016 [87] is a RTLS performance testing and evaluation standard jointly developed by the International Organization for Standardization (ISO) and the International Electrotechnical Commission (IEC). The standard clarifies the test environment, data collection methods, and evaluation process and standardizes key performance indicators, such as the positioning accuracy, response time, stability, and equipment interoperability requirements of the RTLS. However, ISO/IEC 18305 is not applicable to developers or researchers [88].
- ISO/IEC 24730-1:2014 [89] allows application software to utilize real-time location system (RTLS) infrastructures for monitoring individuals or items equipped with RTLS transmitters.
- ISO 19116:2019 [90] outlines the data format and communication protocol for devices that provide and utilize positional information. It proves beneficial in numerous location-centric applications, including navigation, surveying, and location-based services.
- ISO 19133:2005 [91] and ISO 19134:2006 [92] specifications describe data types and operations associated with multimodal tracking and navigation.
- Bluetooth Core Specification version 5.1 [93] introduces a new direction-finding feature. This capability allows Bluetooth gadgets to achieve precision within centimeters.
- The Wi-Fi Round Trip Time (RTT) [94] feature is a positioning technology based on the IEEE 802.11 mc protocol. It calculates the distance by accurately measuring the signal RTT between the device and the Wi-Fi access point to achieve sub-meter positioning. Wi-Fi RTT has the advantages of requiring no additional hardware, supporting multi-access point co-location, not relying on signal strength (RSSI), a stronger anti-interference ability, and good privacy.
- 3GPP Release 13 [95] demonstrated the capability of LTE to fulfill certain indoor positioning requirements and proposed potential improvements. Key enhancements have been formalized in the recently completed 3GPP Release 16 and 17 projects.

### 1.3. Related Competitions

Competitions offer a platform for assessing various positioning algorithms under identical experimental conditions, error metrics, and datasets.

- The Indoor Positioning and Indoor Navigation (IPIN) Competitions [96,97,98] has been organized by the International Conference on Indoor Positioning and Indoor Navigation since 2011. The IPIN Competition includes real-time and post-processing tracks based on smartphones, vision solutions, ultra-wideband (UWB), 5G positioning, micro-inertial navigation systems, etc. The participating teams need to achieve high-precision position tracking in different scenarios, such as factories and vehicle environments. The competition evaluates the positioning accuracy, stability, and environmental adaptability of the positioning system through real-time dynamic testing and static data analysis tasks, provides a reference framework for technology standardization and industrialization, and promotes collaborative innovation between academia and industry.
- The UPINLBS Competition [99] is an event organized by the International Ubiquitous Positioning, Indoor Navigation and Location-Based Services (UPINLBS) Conference. It focuses on indoor navigation and consists of three distinct categories: Bluetooth, UWB, and INS. The competition comprises two phases: an initial round and a concluding round.
- The PerfLoc Prize Competition [100,101] was hosted by the US National Institute of Standards and Technology (NIST) [102] with the aim of gathering a substantial amount of smartphone data from global researchers for the advancement of localization algorithms. The NIST provided a web portal for assessment to evaluate the efficacy of these algorithms, inviting top-performing ones for live testing. The evaluation process adhered to the widely recognized ISO/IEC 18305 standard.
- The Microsoft Indoor Localization Competition [103], organized by IPSN, is an International Conference on Information Processing in Sensor Networks and aims to compare real-time or near real-time indoor positioning technologies based on their performance. Collaboratively conducted by the Microsoft indoor location competition committee and XYZ10, the Indoor Location Competition 2.0 [104] releases a comprehensive dataset from approximately 1000 buildings containing inertial sensors, geomagnetic signals, Bluetooth signals, and Wi-Fi signals along with corresponding ground truths.
- The PDR/xDR Challenges [105] are a series of competitions arranged by the committee responsible for establishing PDR benchmark standards. These contests prioritize practicality and emphasize PDR techniques without requiring specialized facilities.

### 1.4. Challenges in Testing and Evaluation

Currently, the performance testing and evaluation of indoor positioning systems (IPSs) faces many challenges, including technical diversity, complex and changeable test environments, tester subjective factors, and the lack of unified evaluation standards. This article provides a detailed analysis of these challenges as follows:

- Technology comparison challenges: Different indoor positioning systems usually consist of one or more technologies, such as Bluetooth, Wi-Fi, ultra-wideband, visual positioning, and inertial navigation. Each technology has different testing methods and evaluation standards. How to conduct a fair and unbiased performance evaluation of complex and diverse positioning systems is a major challenge. In addition, different positioning system technologies may show different performance differences in different application scenarios. Scenario testing is required to ensure that the performance of each scenario meets expectations.
- Difficulty in data collection and annotation: It is very difficult and costly to obtain high-precision position true values of reference points on a large scale through equipment, such as Vicon [106], OptiTrack [107], in an indoor environment. Annotating the real trajectories of dynamic behaviors, such as human movement and device interaction, introduces potential errors and proves difficult. Additionally, the accuracy of time synchronization among data from multiple sensors (e.g., cameras, IMUs, and wireless signals) significantly impacts the evaluation of fusion algorithms.
- Subjective factors of the tester: It is difficult to ensure that the movement trajectory, tester carrying device mode (handheld, pocket), and movement mode (walking, running) are exactly the same during each test. Different device carrying modes and movement modes have a greater impact on positioning results. Different testers and devices present obvious data heterogeneity. In addition, subjective feelings, such as positioning latency and interface usability, are difficult to measure using purely technical indicators.

### 1.5. Key Contributions and Organization

**Figure 3:**
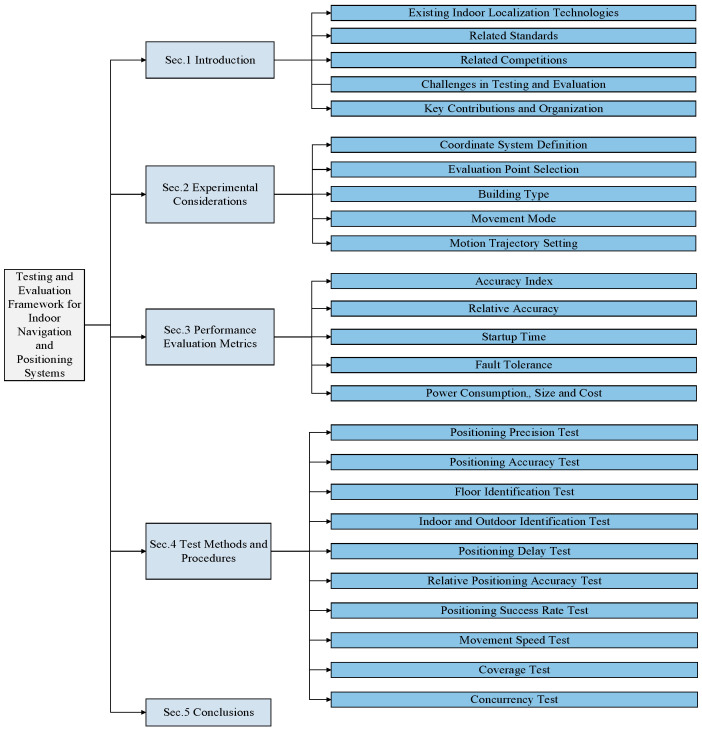
The organization of this paper.

At present, the industry lacks the effective means for an indoor positioning system performance evaluation. For key performance indicators, such as positioning accuracy and positioning delay, domestic and foreign standard organizations and the industry have not yet formed a unified evaluation method. Establishing benchmarking techniques, universal assessment standards, and consistent methodologies that benefit developers, testers, and users remains a critical challenge [88,108,109]. This paper integrates global standards (ISO/IEC 18305) with extensive research experience, covering experimental factors, performance assessment criteria, testing methodologies, and procedures to develop an indoor positioning performance evaluation framework, offering a comprehensive system for testing and evaluating positioning and tracking technologies. Figure 3 illustrates the structure of this study.

## 2. Experimental Considerations

Indoor positioning experimental environments typically encompass several key elements: the coordinate system definition, evaluation point selection, building type classification, movement mode determination, and motion trajectory configuration.

### 2.1. Indoor Coordinate System

Two primary methods exist for establishing an indoor coordinate system: absolute and relative coordinate systems. Conventionally, satellite systems, such as WGS 84, ITRS, GTRF, or CGCS 2000, are utilized to define the evaluation region, which is then converted into the three-axis components of the local Cartesian coordinate framework. All the subsequent testing and evaluation occurs within this local Cartesian coordinate framework. However, establishing an absolute coordinate system presents numerous challenges, particularly when satellite signals are unavailable or geographical information is lacking. The aforementioned four coordinate systems are more suitable for large-scale outdoor spaces with significant variations in longitude and latitude. Indoor positioning typically covers a range of a few hundred meters, where longitudinal and latitudinal changes are minimal, and the height information consistently remains perpendicular to the horizontal plane. Consequently, implementing a relative coordinate system for indoor navigation proves to be both simpler and more reliable. Employing the local Cartesian coordinate system as a reference is practical. The selection criteria for the origin point and axes of a Cartesian coordinate system include the following:

(1) The origin is the geometric center of the test and evaluation range, which is generally the geometry of the building. The X and Y axes are freely chosen in the horizontal plane, usually parallel to the building profile or corridor. The Z axis is perpendicular to the horizontal plane formed by the XY axis and upward.
(2) The coordinate system established must be convenient for surveying and mapping. In practical applications, electronic maps are often combined, and an electronic fence is constructed using maps and building shapes. The relative Cartesian coordinate system is within the fence, and a certain point or surface has a physical meaning, such as representing a room or a certain floor.

### 2.2. Building Type

Different building materials have a great impact on the positioning performance. Generally speaking, different types of buildings are suitable for different positioning technologies (for example, buildings made of non-metallic materials produce less distortion of the magnetic field, resulting in poor positioning performance, and environments such as mines have strict requirements for electromagnetic signals). Table 2 divides the common building types into five categories: Cabin, Brick house, Factory, Skyscraper, and Underground or mine. Each building type is defined based on its structural characteristics, electromagnetic interference level, number of floors, and area size in order to evaluate its impact on the positioning system signal propagation.

### 2.3. Evaluation Point Selection

(1) The true value of the evaluation point is usually its use of offline surveying and mapping. At this time, the accuracy of the surveying and mapping instrument must be at least one order of magnitude higher than the positioning accuracy; or a reference system whose average accuracy is one order of magnitude higher than the test system is selected. The point of the evaluation is to compare the true value point of the survey with the output of the system under test at the true value point, or the measurement difference between the reference system and the system to be evaluated under the same conditions, or simply to refer to the comparison of the post-processing results of the same set of data by different algorithms.

(2) The evaluation points must be different from the training points, and selected non-uniformly (determined by the probability that the area where the evaluation point is located is visited), and sufficient coverage must be ensured according to the minimum accuracy requirements of the system.

(3) Ensure the independence of each assessment point during surveying and mapping. If it is not met, the principle of closed-loop surveying and mapping should be adopted to eliminate compound errors.

(4) Variables should be controlled to achieve repeated measurements and multiple measurements of the system to be evaluated. If the variables are not easy to control, such as the movement speed and movement state (walking, running, jumping, etc.) of the test point and the entity to be localized/tracked, static objects should be considered for testing and evaluation.

(5) The number of test points should be no less than 20.

The basic information on the evaluation points is shown in Table 3. The number of evaluation points in a building correlates with its area. Generally, one test point is selected per 5 to 10 m^2^ on average. At least half of the test points should be evaluated in a single test. All test points should be covered in all tests, and all areas should be treated consistently. Furthermore, when measuring or visualizing the position drift error resulting from dead reckoning positioning, a straight test distance of at least 50 m should be incorporated into the test scenario. During the actual test, it is essential to record the location of the building being tested (typically known), including street information, test time, and environmental data, such as the temperature, humidity, and pressure on the day of testing.

### 2.4. Mobile Mode

For positioning methods based on wireless signal intersection or database matching, the movement pattern of the carrier has almost no impact on the measurement results [111,112,113]. However, the movement mode has a greater impact on the dead reckoning technology based on inertial sensors [114]. The movement pattern is defined here as the typical movement pattern of all the carriers that affect the indoor positioning technology. Carriers include humans, robots, etc. The movement modes [115] include walking, running, moving backward, moving sideways, climbing, going up and down stairs, etc., as shown in Table 4. If the carrier is a human, volunteers need to be recruited and tested in groups based on their average body size. At the same time, a complete test process should include multiple movement modes to test the performance of the system when switching modes.

### 2.5. Motion Trajectory Settings

Common motion trajectories [116,117] encompass the linear, L-shaped, T-shaped, rectangular, and circular paths. Consequently, performance evaluations should incorporate both open-loop trajectories (such as linear, L-shaped, and T-shaped) and closed-loop trajectories (including rectangular and circular paths) [118]. The open-loop trajectory test is to avoid the reduction in the position drift error due to the back propagation due to the closed-loop. The closed-loop trajectory test facilitates the calculation of end-to-end errors and the visualization of the deviation between the positioning endpoint and the closed-loop area. Trajectory settings are often related to the feasible paths of the building being tested. Comparing the visual similarity between the trajectory calculated by the positioning algorithm and the preset trajectory will help quantify the distance deviation between the starting and endpoints.

## 3. Performance Evaluation Metrics

Different companies, different research institutions, and different researchers have different indicators for evaluating RTLSs, making it difficult to compare different RTLSs fairly and impartially [119]. Currently, no single navigation and positioning technique prevails in the market due to the absence of standardized datasets and metrics for comparing and assessing the available positioning solutions [10,27,120]. We refer to the ISO/IEC 18305 standard [87] and use the previous research on testing and evaluating RTLSs to define the performance indicators, such as the accuracy index, relative accuracy, startup time, fault tolerance, power consumption, size, and cost.

### 3.1. Accuracy Index

Table 5 systematically summarizes the multi-dimensional indicators for the RTLS accuracy evaluation, including basic parameters, such as the error vector, error modulus, floor recognition rate, and regional detection probability, as well as statistical characteristics, such as the mean, covariance, and root mean square error (RMSE). At the same time, three types of probability errors (Vertical Error Probable (VEP)/Circular Error Probable (CEP)/Spherical Error Probable (SEP)) and cumulative distribution functions (CDFs) are defined, and the dual standards of the 50% probability error (VEP/CEP/SEP) and 95% probability error (VE95/CE95/SE95) are used as reference indicators.

### 3.2. Relative Accuracy

The relative accuracy refers to the accuracy of measuring the distance between the target position and the reference point in a specific reference system (such as a device group or local environment), emphasizing the relative position relationship in the local space rather than absolute geographical coordinates. It is suitable for scenarios such as firefighting, emergency rescue, drone formation, industrial robot collaborative operations, AR/VR space interaction, etc.

### 3.3. Startup Time

The startup time encompasses the duration required for various preparatory tasks before a system becomes fully operational. These preparations include the offline construction of positioning infrastructure, on-site equipment calibration, manual surveying and mapping, and database development (e.g., for indoor positioning systems utilizing Wi-Fi, RFID, BLE, and other technologies). It also includes the time consumed by online preparations such as the algorithm parameter configuration, preprocessing, parameter initialization, algorithm initial solution convergence, etc. (for example, when the position initialization based on satellite systems often takes more than ten minutes to obtain a stable convergence solution; INS often requires a few minutes of warmup time, also called hot start time, to allow the inertial device to reach a relatively stable working state). It even includes the time to establish the local Cartesian coordinate system and the time to obtain the key parameters of the building (in some applications it is necessary to know the floors, corners, stair entrance, and exit locations of the building and the coordinate information of some corners). The startup time should be clear and detailed based on the specific application so that people can evaluate the complexity of the startup, the speed of the startup, and the cost of the startup. For ultra-fast startup scenarios under non-structural conditions that require the system to work as soon as it is powered on, such as individual combat, fire rescue, etc., the startup time should be controlled within a few seconds.

### 3.4. Fault Tolerance

No positioning system can operate optimally in all temporal and spatial contexts or under every emergency scenario. Consequently, the fault tolerance measures a positioning system’s resilience to unforeseen circumstances within specified time and space parameters. This concept, also referred to as robustness, sensitivity, or flexibility, is closely tied to the specific operational environment and mode. The fault tolerance can be evaluated through a combination of classic and random mode tests. The results of these assessments can then be utilized as feedback to enhance the system fault tolerance, either by incorporating redundant hardware subsystems or by refining and optimizing algorithms.

### 3.5. Power Consumption, Size, and Cost

The power consumption is related to battery life. Low power consumption means a longer working time, which indirectly affects the limit of the positioning area. The volume means portability, miniaturization, and miniaturization are the consistent trends, and a low cost is the prerequisite for mass production and application. Usually, for ordinary positioning systems, accuracy is often given top priority, but from a system engineering perspective, a variety of factors should be considered comprehensively in the early stages of design for a relatively complex positioning system.

## 4. Test Methods and Procedures

The indoor positioning system’s evaluation typically encompasses a range of tests, including positioning precision, accuracy, floor identification, indoor–outdoor distinction, latency, relative accuracy, success rate, and movement speed tests. These comprehensive evaluations ensure the system’s reliability and effectiveness across various performance metrics.

### 4.1. Positioning Precision Test

Positioning precision is used to test the standard deviation of the coordinates of the point to be measured by the positioning system. The test scenario should include the following three scenarios: (a) outdoor space; (b) scene switching between outdoor space and indoor space; and (c) indoor space, which should include open spaces, multiple rooms, basements, and switches between floors, such as stairs, escalators, and straight ladders.

The selection of test points and the true value measurement are described in Section 2.3. A certain proportion of *N* test points are selected from the test points as points to be tested. The testing procedure adheres to the following steps:

(1) Start the system under test;
(2) Perform a static positioning test: The positioning terminal moves along the predetermined test path in the test field. The movement method is shown in Section 2.4. Stop at the point to be tested in the test path and wait for no less than 5 s. According to the position update frequency of the system under test, M coordinate measurement values of the point under test are generated and recorded as $\left(x_{ij},y_{ij},z_{ij}\right)(i=1,2,\cdots ,N,j=1,2,\cdots ,M)$, and then move to the next point to be measured. Repeat the above steps until all N points to be measured are visited;
(3) Calculate the mean value of the positioning coordinates of each point to be measured, according to Equation (1);
  (1) $$\bar{x_{i}}=\frac{\sum_{j=1}^{M}x_{ij}}{M},\;\bar{y_{i}}=\frac{\sum_{j=1}^{M}y_{ij}}{M},\;\bar{z_{i}}=\frac{\sum_{j=1}^{M}z_{ij}}{M}$$
(4) Calculate the standard deviation of the positioning coordinates of each point to be measured according to Equation (2);
  (2) $$\sigma_{xi}=\sqrt{\frac{[\sum_{j=1}^{M}\left(x_{ij}-\bar{x_{i}}{)}^{2}\right]}{M}},\sigma_{yi}=\sqrt{\frac{[\sum_{j=1}^{M}\left(y_{ij}-\bar{y_{i}}{)}^{2}\right]}{M}},\sigma_{zi}=\sqrt{\frac{[\sum_{j=1}^{M}\left(z_{ij}-\bar{z_{i}}{)}^{2}\right]}{M}}$$
(5) Calculate the positioning accuracy of each measured point in sequence according to Equation (3);
  (3) $$\sigma_{ri}=\sqrt{{\sigma_{xi}}^{2}+{\sigma_{yi}}^{2}+{\sigma_{zi}}^{2}}$$
(6) Take the maximum value $\sigma_{r,max}$ of $\sigma_{ri}$ as the positioning accuracy of the system;
(7) Shut down the system under test.

### 4.2. Positioning Accuracy Test

Positioning accuracy is used to test the standard deviation of the coordinates of the point to be measured by the positioning system. The test scenario should include the following three scenarios: (a) outdoor space; (b) scene switching between outdoor space and indoor space; and (c) indoor space, which should include open spaces, multiple rooms, basements, and switches between floors, such as stairs, escalators, and straight ladders.

The selection of test points and the true value measurement are described in Section 2.3. A certain proportion of N test points are selected from the test points as points to be tested. The testing procedure adheres to the following steps:

(1) Start the system under test;
(2) Conduct two sets of tests: the static positioning test and dynamic positioning test:
  (a) Static positioning test: The positioning terminal moves along the predetermined test path in the test field. The movement method is shown in Section 2.4. It stops at the point to be tested in the test path, waits for no less than 5 s, and updates according to the position of the system under test. For the frequency, generate M coordinate measurement values of the point to be measured, recorded as $\left(x_{ij},y_{ij},z_{ij}\right)(i=1,2,\cdots ,N,j=1,2,\cdots ,M)$, and then move to the next points to be tested, repeat the testing method in this column until all N points to be tested are visited;
  (b) Dynamic positioning test: The positioning terminal moves at a constant speed along the predetermined test path in the test field. The movement method is shown in Section 2.4. When moving to the point to be tested on the test path, the position coordinates of the point to be tested are generated and recorded as $\left(x_{i},y_{i},z_{i}\right)(i=1,2,\cdots ,N)$, then move to the next point to be tested, and repeat the testing method of this column until all N points to be tested are completed.
(3) Calculate the positioning distance error of the static positioning test;
  (a) Calculate the mean value of the positioning coordinates of each point to be measured according to Equation (1);
  (b) Calculate the positioning error coordinates of each point to be measured according to Equation (4):
    (4) $$\varepsilon_{i}=(\Delta x_{i},\Delta y_{i},\Delta z_{i})=(\Lambda x_{i}-\bar{x_{i}},\Lambda y_{i}-\bar{y_{i}},\Lambda z_{i}-\bar{z_{i}})$$
  (c) Calculate the positioning error between the positioning coordinates of each measured point and the real coordinates according to Equation (5):
    (5) $$\left|\varepsilon_{i}\right|=\sqrt{\Delta x_{i}^{2}+\Delta y_{i}^{2}+\Delta z_{i}^{2}}$$
(4) Calculate the positioning error for the dynamic positioning tests:
  (a) Calculate the positioning error coordinates of each point to be measured according to Equation (6):
    (6) $$\varepsilon_{i}=(\Delta x_{i},\Delta y_{i},\Delta z_{i})=(\Lambda x_{i}-x_{i},\Lambda y_{i}-y_{i},\Lambda z_{i}-z_{i})$$
  (b) Calculate the positioning error between the positioning coordinates of each measured point and the real coordinates according to Equation (5);
(5) Calculate the circular probability error or spherical probability error according to Equation (7):
  (7) $$\begin{array}{c} SEP\alpha =min\{R:R≧0,|\{\varepsilon_{i}:i=1,2,\ldots ,N;|\varepsilon_{i}|≦R\}|≧0.01*\alpha N\}\\ CEP\alpha =min\{R:R≧0,|\{\varepsilon_{i}:i=1,2,\ldots ,N;|\varepsilon_{i}|≦R\}|≧0.01*\alpha N\} \end{array}$$
(6) Use the probability error as the positioning accuracy of the system under test;
(7) Shut down the system under test.

Note 1: the common value of a is 50 or 95; that is, SEP95 is a 95% ball probability error, SEP50 is a 50% ball probability error, and the same is true for CEP.

$$\mathrm{Example}\;{SEP}_{95}=min\{R:R⩾0,|\{\varepsilon_{i}:i=1,2,\cdots ,N;|\varepsilon_{i}|\;⩽R\}|\;⩾0.01\times 95\times N\}$$

Note 2: $|\{\varepsilon_{i}:i=1,2,\cdots ,N;|\varepsilon_{i}|⩽R\}|⩾0.95\times N$ means that the number of elements in the set composed of positioning errors smaller than R is not less than 0.95×N. The ${SEP}_{95}$ value is the minimum value that can be greater than any non-repeating 0.95×N or more values in the horizontal positioning error value set. The meaning of ${SEP}_{95}$ is that 95% and above of the positioning test points fall within the sphere with the real coordinates as the center of the sphere and ${SEP}_{95}$ as the minimum radius.

### 4.3. Relative Positioning Accuracy Test

This indicator is used to test the relative position accuracy between the two sets of coordinates of the points to be measured by the positioning system. The test scenario should include the following three scenarios: (a) outdoor space; (b) scene switching between outdoor space and indoor space; and (c) indoor space, which should include open spaces, multiple rooms, basements, and switches between floors, such as stairs, escalators, and straight ladders.

The selection of test points and the true value measurement are described in Section 2.3. Select a certain proportion of 2*N* test points from the test points and divide them into two groups, with the N test points in each group as the points to be tested in each group. The testing procedure adheres to the following steps:

(1) Start the system under test;
(2) Perform a dynamic positioning test: Two positioning terminals move along two predetermined sets of different test paths in the test field. The movement method is shown in Section 2.4. When moving to the point to be tested on the test path, they generate corresponding images of the point to be tested. Position coordinates are the position coordinates of the first positioning terminal at the point to be measured, recorded as $P_{i1}=\left(x_{i1},y_{i1},z_{i1}\right)(i=1,2,\cdots ,N)$, and the position of the second positioning terminal at the point to be measured. The coordinates are recorded as $P_{i2}=\left(x_{i2},y_{i2},z_{i2}\right)(i=1,2,\cdots ,N)$; then, move to the next point to be tested, and repeat the testing method of this column until all N points to be tested are completed;
(3) Calculate the distance between the two positioning terminals at the positioning coordinates of their respective points to be measured according to Equation (8):
  (8) $$r_{i}=\sqrt{(x_{i1}-x_{i2}{)}^{2}+(y_{i1}-y_{i2}{)}^{2}+(z_{i1}-z_{i2}{)}^{2}}$$
(4) Calculate the relative accuracy calculated according to Equation (9). This metric is represented by “$\mu_{\Delta }$” and is defined as the average of the absolute difference between the distance measured by two positioning terminals measured simultaneously and the true distance;
  (9) $$\mu_{\Delta }=\sum_{i=1}^{N}\left|\Lambda r_{i}-r_{i}\right|/N$$
(5) Shut down the system under test.

### 4.4. Floor Identification Test

This indicator is used to test the floor judgment accuracy of the coordinates of the point to be measured by the indoor positioning system. The test scene is an indoor space, which should include switching between floors, such as the starting and reaching positions of stairs, escalators, and elevators. In order to avoid ambiguity in the floor determination, avoid selecting the position in the middle of two floors of stairs or the position above an escalator or straight ladder as a test point.

The selection of test points and the true value measurement are described in Section 2.3 A certain proportion of N test points are selected from the test points as points to be tested. The testing procedure adheres to the following steps:

(1) Start the system under test;
(2) Conduct two sets of tests: the static positioning test and dynamic positioning test:
  (a) Static positioning test: The positioning terminal moves along the predetermined test path in the test field. The movement method is shown in Section 2.4. It stops at the point to be tested in the test path, waits for no less than 5 s, and updates according to the position of the system under test. For the frequency, generate the M floor number measurement results $F_{ij}(i=1,2,\cdots ,N,j=1,2,\cdots ,M)$ of the point to be measured, then move to the next point to be measured, and repeat the steps of this column test method until all N points to be tested are completed;
  (b) Dynamic positioning test: The positioning terminal moves at a constant speed along the predetermined test path in the test field. The movement method is shown in Section 2.4. When moving to the point to be measured on the test path, generate the floor number measurement result $F_{i}(i=1,2,\cdots ,N)$ of the point, then move to the next point to be measured, and repeat the test method of this column until you complete all N points to be tested;
(3) Calculate the correctness of the measurement results of each floor number in sequence according to Equation (10):
  (10) $$\Delta F=\left\{\begin{array}{l} \begin{array}{cc} 1 & The\;floor\;number\;is\;correct \end{array}\\ \begin{array}{cc} 0 & The\;floor\;number\;is\;incorrect \end{array} \end{array}\right.$$
(4) Calculate the floor judgment accuracy of the static positioning test according to Equation (11):
  (11) $$FR=\sum_{i=1}^{N}\sum_{j=1}^{M}\Delta F_{ij}/(M\times N)$$
(5) Calculate the floor judgment accuracy of the dynamic positioning test according to Equation (12):
  (12) $$FR=\sum_{i=1}^{N}\Delta F_{i}/N$$
(6) Shut down the system under test.

### 4.5. Indoor and Outdoor Identification Test

This indicator is used to test the indoor and outdoor judgment accuracy of the coordinates of the point to be measured by the positioning system. The test scene is the scene switching between the outdoor space and indoor space, such as walking in or out of the building through the building door. A certain proportion of N test points are selected from the test points as points to be tested. The testing procedure adheres to the following steps:

(1) Start the system under test;
(2) Conduct indoor and outdoor judgment tests: the positioning terminal triggers a positioning request at the first point to be measured, generates the indoor and outdoor IO value measurement results of the point to be measured, recorded as $IO_{i}(i=1,2,\cdots ,N)$, and then moves to the next point to be tested, and repeat the testing method in this column until all N points to be tested are completed;
(3) Calculate the correctness of each indoor and outdoor value measurement result in sequence according to Equation (13):
  (13) $$\Delta IO=\left\{\begin{array}{cc} 1 & Correct\;judgment\\ 0 & Incorrect\;judgment \end{array}\right.$$
(4) Calculate the indoor and outdoor recognition accuracy according to Equation (14):
  (14) $$IOR=\sum_{i=1}^{N}\Delta IO_{i}/N$$
(5) Shut down the system under test.

### 4.6. Positioning Delay Test

This indicator is used to test the time difference between the positioning server receiving the positioning request and the positioning server calculating the test point location information. The test scenario should include the following three scenarios: (a) the outdoor space; (b) scene switching between the outdoor space and indoor space; and (c) indoor space, which should include open spaces, multiple rooms, basements, and switches between floors, such as stairs, escalators, and straight ladders.

The selection of test points and true value measurement are described in Section 2.3. A certain proportion of N test points are selected from the test points as points to be tested. The testing procedure adheres to the following steps:

(1) Start the system under test;
(2) Perform the positioning delay test:
  (a) The positioning terminal triggers a positioning request at the first point to be measured, records the time when the positioning server receives the calculated position coordinates of the point to be measured, and records the trigger time $T_{i1}$;
  (b) Record the time $T_{i2}$ when the positioning server gives the position of the positioning terminal;
  (c) Then, move to the next point to be measured until all N points to be measured are completed.
(3) Calculate the positioning delay of each point to be measured according to Equation (15):
  (15) $$\Delta T_{i}=T_{i2}-T_{i1}$$
(4) Take the maximum value $\Delta T_{i,max}$ of $\Delta T_{i}$ as the positioning delay of the system;
(5) Shut down the system under test.

### 4.7. Positioning Success Rate Test

The positioning success rate is used to test the positioning system. When multiple positioning terminals are positioned simultaneously, the system under test can give the success rate of the positioning terminal results. It is measured by the ratio of the number of terminals that give positioning results to the total number of positioning terminals. The test method is as follows:

(1) Select N test points as points to be tested;
(2) Start the positioning system;
(3) Place a terminal on all test points and conduct static positioning tests. The number of positioning times is P. Calculate and record the time $T_{ip}(i=1,2,\cdots ,N;p=1,2,\cdots ,P)$ when the server gives the position of each positioning terminal;
(4) According to Equation (16), calculate the time difference between the time when each positioning terminal tests the positioning server to give the positioning terminal position and the time when the positioning server gives the positioning terminal position in the last test:
  (16) $$\Delta T_{ip}=T_{ip}-T_{i(p-1)}$$
(5) According to Equation (17), calculate the number of terminals for which the system under test gives terminal positioning results in each test:
  (17) $$\begin{array}{c} \Delta L_{ip}=\left\{\begin{array}{cc} 1 & (\Delta T_{ip}<1.5\times Location\;update\;frequency\\ 0 & (\Delta T_{ip}>1.5\times Location\;update\;frequency \end{array}\right.\\ L_{p}=\sum_{i=1}^{N}\Delta L_{ip} \end{array}$$
(6) According to Equation (18), calculate the average number of terminals that provide positioning results for each tested system in P positioning tests:
  (18) $$\bar{L}=\sum_{p=2}^{P}L_{p}/(P-1)$$
(7) Calculate the system positioning success rate according to Equation (19):
  (19) $$S=\bar{L}/N$$
(8) Shut down the system under test.

### 4.8. Movement Speed Test

The moving speed is used to test whether the positioning results of the positioning system meet the requirements at the nominal terminal displacement speed. The test method is as follows:

(1) Select N test points as points to be tested;
(2) Start the positioning system;
(3) The positioning terminal passes the test point at the nominal moving speed in the test field, and the positioning accuracy and positioning success rate of the terminal at the test point are calculated and recorded;
(4) Compare the positioning accuracy of each test point with the nominal positioning accuracy value given by the system to determine whether it is greater than the nominal value given by the system. Compare the positioning success rate of all test points with the positioning success rate given by the system. Compare the rate to determine whether it is less than the nominal value given by the system;
(5) If the positioning accuracy of no test point is greater than the nominal positioning accuracy value given by the system and the positioning success rate of all test points is greater than the positioning success rate given by the system, then the nominal moving speed of the system under test meets the requirements;
(6) Shut down the system under test.

### 4.9. Coverage Test

The coverage test is used to test whether the positioning result of the positioning system meets the requirements within the nominal positioning equipment coverage. The test method is as follows:

(1) Select N test points in a full coverage manner within the nominal coverage range of the positioning system;
(2) Start the positioning system;
(3) Place one terminal on each point to be tested, conduct a static positioning test, and calculate and record the positioning accuracy and positioning success rate of the terminal at the point to be tested;
(4) Compare the positioning precision and positioning accuracy of each point to be measured with the positioning precision and positioning accuracy nominal values given by the system, determine whether it is greater than the nominal value given by the system, and determine whether the positioning of all test points is successful. Compare the positioning success rate with the positioning success rate given by the system to determine whether it is less than the nominal value given by the system;
(5) If the positioning precision and accuracy of all test points do not exceed the nominal values provided by the system, and the positioning success rate at each test point surpasses the system-provided rate, then the nominal coverage of the system under test meets the requirements;
(6) Shut down the system under test.

### 4.10. Concurrency Test

The concurrency is used to test whether the positioning results of the RTLS meet the requirements within the nominal terminal concurrency. The test method is as follows:

(1) Select N test points within the nominal coverage of the positioning system. The number of test points is equal to the nominal concurrency of the system under test;
(2) Start the positioning system;
(3) Place a terminal on all test points, conduct static positioning tests, calculate and record the positioning precision, positioning accuracy, and positioning success rate of all terminals at the corresponding test points;
(4) Compare the positioning precision and positioning accuracy of each test point with the nominal value of the positioning precision and positioning accuracy given by the system, determine whether it is greater than the nominal value given by the system, and compare the positioning success rate of all test points. Compare with the positioning success rate given by the system to determine whether it is less than the nominal value given by the system;
(5) If the positioning accuracy and positioning precision of no test points are greater than the nominal positioning precision and positioning accuracy values given by the system and the positioning success rate of all test points is greater than the positioning success rate given by the system, then the measured nominal concurrency of the system meets the requirements;
(6) Shut down the system under test.

## 5. Conclusions

After more than 20 years of development, indoor positioning technology has become more mature and has begun to enter the promotion and application stage. However, the lack of effective performance testing and evaluation methods is one of the obstacles hindering the popularization and application of indoor positioning technology. Specifically, the test environments, datasets, performance indicators, and testing processes of different positioning technologies are different, and there are no clear and unified specifications, making it difficult to objectively and fairly reflect the positioning accuracy, stability, and environmental adaptability of different positioning technologies.

This article combines international standards (ISO/IEC 18305) and the China Electronics Standardization Institute’s many years of standard setting experience in real-time positioning to build a testing and evaluation framework for indoor navigation and positioning systems. It covers experimental considerations, performance evaluation metrics, test methods, and procedures for building an indoor positioning performance evaluation system. This paper provides a comprehensive framework for performance testing and evaluation for navigation and positioning system design, development, and testing personnel and provides effective guidance for the testing, inspection, or certification services for real-time positioning system products.

## Figures and Tables

**Table 1:** Comparison of typical indoor positioning technologies.

Positioning Technology	Positioning Principle	Representative System	Positioning Accuracy	Advantage	Shortcoming
GNSS	Multilateration	GPS, Beidou	cm–100 m	Widely available	Indoor non-availability; multipath effect
Infrared	Using infrared and receiver, based on geometric measurement principle	Active Badge [68]	Up to millimeter level	High positioning accuracy, compact equipment, easy system installation	Requires additional hardware, requires line-of-sight transmission, and is easily affected by sunlight, etc.
Ultrasound	Using ultrasonic arrival time, based on geometric measurement principle	Active Bat [73]	Up to centimeter level	High positioning accuracy and simple system structure	The hardware cost is high, the signal transmission attenuation is obvious, and the positioning range is affected
RFID	Using the time difference in arrival of radio frequency signals, based on the principle of geometric measurement	LANDMARC [71], SpotON	Average error in 2–3 m	Non-line-of-sight transmission, small hardware size and low cost	Complex system installation
Bluetooth	Using signal strength, based on signal propagation models or fingerprints	iBeacon [74], TOPZ [75]	Average error in meters	Non-line-of-sight transmission, low power consumption and small size	The signal transmission distance is short and the stability is poor
Ultra-Wideband	By transmitting and receiving ultra-wideband pulse signals, based on the geometric measurement principle	Ubisense [70]	Up to centimeter level	High positioning accuracy, strong signal penetration, and good anti-multipath effect	The hardware cost is high and the technology is not mature enough
Wireless Sensor Networks	Using network node signal distance measurement or network connectivity	Zigbee	Average error in meters	Non-line-of-sight transmission, low energy consumption	Applied to specific fields, network stability and robustness need to be improved
Wi-Fi	Using signal strength, based on signal propagation models or fingerprints	RADAR [76], Horus [77], Nibble [78]	3–10 m	No additional equipment required, low cost, wide range of applications	Signal fluctuation characteristics, large workload of fingerprint collection
Wi-Fi FTM/RTT	Calculates distance via Round-Trip Time (RTT) measurements and triangulates using multiple APs	TimeSense [79], WiNar [80], MOC [81]	1–3 m	No fingerprint database, better multipath resistance	Hardware dependent (802.11 mc), High AP density required
Wi-Fi CSI	Analyzes multipath characteristics and environmental changes using Channel State Information (phase/amplitude).	DeepFi [82], D-Fi [83]	0.1–1 m	Ultra-high precision, strong environmental robustness, supports behavior sensing (e.g., gesture recognition)	High computational complexity, strict hardware requirements (e.g., Intel 5300 NIC)
Visible light	Using optical signal intensity or imaging techniques, based on geometric measurements or fingerprinting	YellowDot, Bytelight	Up to centimeter level	High positioning accuracy, low energy consumption, and free from electromagnetic interference	Requires line-of-sight transmission, complex system deployment, high cost, and immature technology
Optics and Vision	Based on image matching and scene analysis	QR-based Navigation, Easy Living	Centimeter level	High positioning accuracy, free from electromagnetic interference	High complexity, high power consumption, easily affected by light intensity, obstruction by obstacles, etc.
Geomagnetic	Fingerprint matching	LocateMe [84], IndoorAtlas [72]	Up to 2–6 m	Geomagnetic signals are ubiquitous and relatively stable, and no additional hardware is required	The geomagnetic fingerprint data dimension is insufficient, the fingerprint collection workload is large, and it is easily affected by metal objects.
Pedestrian Dead Reckoning (PDR)	Using inertial sensor data, based on the principle of inertial navigation	SmartPDR [85]	Can reach meter level	Simple, low-cost, not affected by electromagnetic signals	An initial position is required, and sensor error accumulation causes position drift
Base Station Positioning	Calculating the current location using the communication time difference between the base station and the mobile phone	E-911 [86]	Ten meters to several hundred meters	No additional equipment is required, relying on mobile phone hardware positioning	Low accuracy and poor reliability
Pseudolite	Place transmitters on the ground that emit signals similar to GPS to enhance or replace satellite signals	SnapTrack	Centimeter level	High positioning accuracy and fast positioning speed	High equipment cost

**Table 2:** Typical building types for indoor positioning systems [110].

Building Type	Definition
Cabin	No electromagnetic interference, the simplest test environment requires an area no less than 200 m^2^.
Brick house	Brick + concrete buildings will affect signal propagation and require at least three floors, with the area of each floor no less than 2000 m^2^.
Factory	Heavy machinery + steel ceiling structure, which will affect signal transmission, single layer, height no less than 5 m, area no less than 5000 m^2^.
Skyscraper	It is a steel + concrete structure with a minimum of 10 floors above ground and may have underground floors, with the area of each floor no less than 1000 m^2^.
Underground or mine	At least 6 m from the surface and an area no less than 2500 m^2^.

**Table 3:** Basic information about assessment points.

Assessment Point	Mathematical Expression	Definition
Position true value (reference value)	$\left(\begin{array}{ccc} x_{i} & y_{i} & z_{i} \end{array}\right)$	N represents a finite natural number, i∈[1,N] represents the i−th reference value.
Estimated location	$\left(\begin{array}{ccc} \hat{x_{i}} & \hat{y_{i}} & \hat{z_{i}} \end{array}\right)$	$i\in \left[1,N\right]\;$represents the *i*-th estimated value.
Floor number	F	There are F floors in total, including underground floors. F represents a natural number.
Floor	j	Indicates the floor, j $\in \left[1,N\right]$ is the serial number of the floor.
Number of areas	$L_{j}$	Use the idea of grid division to divide the same floor into $L_{j}$ areas.
Floor area	$S_{j,1},S_{j,2},\ldots ,S_{j,L_{j}}$	Represents each area of j floor.

**Table 4:** Mobile mode.

Mobile Mode	Definition
Still	Reference value 30 min
Walking	Reference speed 1.2 m/s
Walking slowly	Reference speed 0.25 m/s
Running	Reference speed 2.5 m/s
Jogging	Reference speed 1.8 m/s
Back	Reference speed 0.5 m/s
Side shift	Reference speed 0.75 m/s
Climbing	Reference speed 0.1 m/s
Up and down stairs	Reference speed 1.0 m/s

**Table 5:** Accuracy index.

Index	Mathematical Expression	Definition
Error vector	$\varepsilon_{i}=(\begin{array}{ccc} \varepsilon_{x,i} & \varepsilon_{y,i} & \varepsilon_{z,i} \end{array})=(\begin{array}{ccc} {\hat{x}}_{i}-x_{i} & {\hat{y}}_{i}-y_{i} & {\hat{z}}_{i}-z_{i} \end{array})$ $\varepsilon_{h,i}=\left(\begin{array}{cc} \varepsilon_{x,i} & \varepsilon_{y,i} \end{array}\right),$ $\varepsilon_{z,i}=(\varepsilon_{z,i})$	$\varepsilon_{i}$, $\varepsilon_{h,i}$, and $\varepsilon_{z,i}$ represent the 3-dimensional position error, horizontal error, and vertical error vector, respectively.
Error modulus	$∥\varepsilon_{i}∥\;=\;\sqrt{\varepsilon_{x,i}^{2}+\varepsilon_{y,i}^{2}+\varepsilon_{z,i}^{2}},$ $∥\varepsilon_{h,i}∥\;=\;\sqrt{\varepsilon_{x,i}^{2}+\varepsilon_{y,i}^{2}},$ $∥\varepsilon_{z,i}∥\;=\;|\varepsilon_{z,i}|$	The 3D/horizontal position errors use L2 norm; vertical error uses absolute value.
Floor recognition rate	$P_{F}=\frac{N_{F}}{N}$	There were $N_{F}$ times of correct identification among N times of floor judgments.
Area detection probability	$P_{Z|F}=\frac{N_{F,Z}}{N_{F}}$	Probability of area detection success given correct floor identification.
Means	$\mu_{\varepsilon }=\frac{1}{N}\sum_{i=1}^{N}\varepsilon_{i},$ $\mu_{\varepsilon }=\frac{1}{N}\sum_{i=1}^{N}∥\varepsilon_{i}∥$, $\mu_{\varepsilon_{h}}=\frac{1}{N}\sum_{i=1}^{N}∥\varepsilon_{h,i}∥,$ $\mu_{\left|\varepsilon_{z}\right|}=\frac{1}{N}\sum_{i=1}^{N}|\varepsilon_{z,i}|$	Represents the initial offset and measures the initial position error.
Covariance	$K_{\varepsilon }=cov(\varepsilon )=\frac{1}{N}\sum_{i=1}^{N}{\left(\varepsilon_{i}-\mu_{\varepsilon }\right)}^{T}(\varepsilon_{i}-\mu_{\varepsilon })$	The offset is eliminated. This is an important indicator of system accuracy.
Variances	$\sigma_{\varepsilon }^{2}=\frac{1}{N}\sum_{i=1}^{N}{\left(∥\varepsilon_{i}∥\;-\;\mu_{∥\varepsilon ∥}\right)}^{2},$ $\sigma_{\left\|\varepsilon_{h}\right\|}^{2}=\frac{1}{N}\sum_{i=1}^{N}{\left(∥\varepsilon_{h,i}∥\;-\;\mu_{\left\|\varepsilon_{n}\right\|}\right)}^{2}$, $\sigma_{|\varepsilon_{z}|}^{2}=\frac{1}{N}\sum_{i=1}^{N}{\left(|\varepsilon_{z,i}|\;-\;\mu_{|\varepsilon_{z}|}\right)}^{2}$	Measures the dispersion of errors.
RMSE	$\varepsilon_{x,RMS}=\sqrt{\frac{1}{N}\sum_{i=1}^{N}\varepsilon_{x,i}^{2}},$ $\varepsilon_{y,RMS}=\sqrt{\frac{1}{N}\sum_{i=1}^{N}\varepsilon_{y,i}^{2}},$ $\varepsilon_{h,RMS}=\sqrt{\varepsilon_{x,RMS}^{2}+\varepsilon_{y,RMS}^{2}},$ $\varepsilon_{z,RMS}=\sqrt{\frac{1}{N}\sum_{i=1}^{N}\varepsilon_{z,i}^{2}},$ $\varepsilon_{RMS}=\sqrt{\varepsilon_{h,RMS}^{2}+\varepsilon_{z,RMS}^{2}}$	Measures the deviation between the estimated value and the true value.
Absolute mean	$\mu_{|\varepsilon |}=\frac{1}{N}\sum_{i=1}^{N}|\varepsilon_{i}|=\frac{1}{N}\sum_{i=1}^{N}\left(|\varepsilon_{x,i}|,|\varepsilon_{y,i}|,|\varepsilon_{z,i}|\right)$	Measuring the magnitude of the error modulus
VEP	$VEP=min\{V:V⩾0,\{\{\varepsilon_{Z,i}:i=1,2,\cdots ,N,∥\varepsilon_{Z,i}∥⩽V\}|⩾0.50N\}$ $VE95=min\{V:V⩾0,|\{\varepsilon_{Z,i}:i=1,2,\cdots ,N,∥\varepsilon_{Z,i}∥⩽V\}|⩾0.95N\}$	VEP/CEP/SEP define vertical/horizontal/3D error bounds (50% quantiles) in distance space; VE95/CE95/SE95 represent 95% probability thresholds.
CEP	$CEP=\min\left\{R:R⩾0,|\left\{\varepsilon_{h,i}:i=1,2,\cdots ,N,\varepsilon_{h,i}⩽R\right\}|⩾0.50N\right\}$ $\mathrm{CE}95=\min\left\{R:R⩾0,|\{\varepsilon_{h,i}:i=1,2,\cdots ,N,\varepsilon_{h,i}⩽R\}|⩾0.95N\right\}$	
SEP	$SEP=min\{R:R⩾0,|\{\varepsilon_{i}:i=1,2,\cdots ,N,\varepsilon_{i}⩽R\}|⩾0.50N\}$ $SE95=min\{R:R⩾0,|\{\varepsilon_{i}:i=1,2,\cdots ,N,\varepsilon_{i}\leq R\}|⩾0.95N\}$	
CDF	$\left\{\varepsilon_{i}:i=1,2,\cdots ,N,sort\left(\varepsilon_{i},ascending\right)\right\}$ $\left\{\varepsilon_{k}:k=1,2,\cdots ,N,\varepsilon_{k}⩽\varepsilon_{k+1}\right\}$ $CDF(\varepsilon_{k})=\frac{num\cdot k}{N}\left\{\begin{array}{c} num=1,∥\varepsilon_{k}∥<∥\varepsilon_{k+1}∥\\ num=k^{\prime }-k+1,∥\varepsilon_{k}∥=∥\varepsilon_{k^{\prime }}∥\neq ∥\varepsilon_{k^{\prime }+1}∥ \end{array}\right\}$	CDF plots ascending error magnitudes vs. cumulative probabilities; 0.5/0.95 quantiles indicate 50%/95% error bound.

## Data Availability

Data are contained within the article.
